# Supplementary material for: Modelling Skylarks (Alauda arvensis) to Predict Impacts of Changes in Land Management and Policy: Development and Testing of an Agent-Based Model
Source: PLoS One. 2013 Jun 6;8(6):e65803. doi: 10.1371/journal.pone.0065803 (PMC3675089; doi:10.1371/journal.pone.0065803)
Supplement: Supporting Information S4 — The skylark ODdox as a zipped archive. (ZIP) [file pone.0065803.s004.zip › Skylark_ODdox/struct_diffusor.html]

ALMaSS Skylark ODdox: Diffusor Struct Reference


ALMaSS Skylark ODdox
 2.0


- Main Page
- Related Pages
- Classes
- Files

- Class List
- Class Index
- Class Hierarchy
- Class Members

Public Member Functions |
Public Attributes

Diffusor Struct Reference

`#include <pesticide.h>`

List of all members.

|  |  |
| --- | --- |
| Public Member Functions | |
|  | Diffusor (int a\_dx, int a\_dy, double a\_fraction) |

|  |  |
| --- | --- |
| Public Attributes | |
| int | m\_dx |
| int | m\_dy |
| double | m\_fraction |

---

## Constructor & Destructor Documentation

|  |  |  |  |
| --- | --- | --- | --- |
| Diffusor::Diffusor | ( | int | *a\_dx*, |
|  |  | int | *a\_dy*, |
|  |  | double | *a\_fraction* |
|  | ) |  |  |

inline

References m\_dx, m\_dy, and m\_fraction.

{

m\_dx = a\_dx;

m\_dy = a\_dy;

m\_fraction = a\_fraction;

}

---

## Member Data Documentation

int Diffusor::m\_dx

Referenced by Diffusor().

int Diffusor::m\_dy

Referenced by Diffusor().

double Diffusor::m\_fraction

Referenced by Diffusor().

---

The documentation for this struct was generated from the following file:

- pesticide.h


- Diffusor
- Generated on Thu Jan 10 2013 13:15:36 for ALMaSS Skylark ODdox by
   1.8.1.1
